# Augmentation of osteochondral repair with hyperbaric oxygenation: a rabbit study

**DOI:** 10.1186/1749-799X-5-91

**Published:** 2010-12-06

**Authors:** Alvin Chao-Yu Chen, Mel S Lee, Song-Shu Lin, Leou-Chuan Pan, Steve Wen-Neng Ueng

**Affiliations:** 1 Department of Orthopaedic Surgery, Chang Gung Memorial Hospital & Chang Gung University; 5, Fu-Hsin St., Kweishan, Taoyuan 333, Taiwan, Republic of China; 2 Department of Pathology, Chang Gung Memorial Hospital & Chang Gung University; 5, Fu-Hsin St., Kweishan, Taoyuan 333, Taiwan, Republic of China; 3 Office of the Vice-superintendent, Chang Gung Memorial Hospital & Chang Gung University; 5, Fu-Hsin St., Kweishan, Taoyuan 333, Taiwan, Republic of China

## Abstract

**Background:**

Current treatments for osteochondral injuries often result in suboptimal healing. We hypothesized that the combination of hyperbaric oxygen (HBO) and fibrin would be superior to either method alone in treating full-thickness osteochondral defects.

**Methods:**

Osteochondral repair was evaluated in 4 treatment groups (control, fibrin, HBO, and HBO+fibrin groups) at 2-12 weeks after surgical injury. Forty adult male New Zealand white rabbits underwent arthrotomy and osteochondral surgery on both knees. Two osteochondral defects were created in each femoral condyle, one in a weight-bearing area and the other in a non-weight-bearing area. An exogenous fibrin clot was placed in each defect in the right knee. Left knee defects were left empty. Half of the rabbits then underwent hyperbaric oxygen therapy. The defects in the 4 treatment groups were then examined histologically at 2, 4, 6, 8, and 12 weeks after surgery.

**Results:**

The HBO+fibrin group showed more rapid and more uniform repair than the control and fibrin only groups, but was not significantly different from the group receiving HBO alone. In the 2 HBO groups, organized repair and good integration with adjacent cartilage were seen at 8 weeks; complete regeneration was observed at 12 weeks.

**Conclusions:**

HBO significantly accelerated the repair of osteochondral defects in this rabbit model; however, the addition of fibrin produced no further improvement.

## Background

Successful repair of full-thickness defects in articular cartilage has been a difficult goal to achieve. Spontaneous repair often fails to completely fill the defect and the new tissue is composed of fibrocartilage rather than the superior hyaline cartilage [1,2]. Although cartilage grafts are composed of hyaline cartilage, they may not bond well to the normal cartilage surrounding the injured area [1,3]. Mesenchymal stem cells [4] or chondrocytes loaded on a porous scaffold have been successfully used for repair [5]; however, this technique involves harvesting and culturing cells. It is thus time-consuming and must be done on an individual basis [6-8]. Growth factors have also been used to increase the regeneration and differentiation of chondrocytes [5,8-11]. However, the delivery of growth factors is not site-specific, and the treatment is expensive. Therefore, we require a better understanding of methods to stimulate the growth and improve the quality of regenerating cartilage [12-14].

Exogenous fibrin clots have been used to facilitate healing in canine and equine knee joints [15-17]. Such clots might promote faster and more organized repair of osteochondral defects. Hyperbaric oxygen (HBO) therapy, ie, the intermittent introduction of 100% oxygen in a closed chamber with a pressure of 1 to 3 standard atmospheres, has been successfully used to enhance wound healing, and has been shown, in both clinical and basic studies, to stimulate collagen formation and neovascularization in damaged tissues [13,18,19]. Because both these interventions improve wound healing, but do so by different mechanisms, we hypothesized that the combined use of a fibrin clot as a scaffold and hyperbaric oxygen to stimulate collagen synthesis and neovascularization might result in faster repair and histologically superior cartilage in full-thickness cartilage defects in rabbit knee joints, as compared with spontaneous repair.

## Methods

The study was designed to compare 4 methods of repairing full-thickness cartilage defects: no treatment, fibrin alone, HBO alone, and HBO plus fibrin. All the authors certify that our institution has approved the animal protocol for this investigation and that all investigations were conducted in conformity with the principles of ethical research. Table 1 shows the study design. Forty rabbits with identical cartilage defects in each knee had fibrin clots placed in the defects in their right knees; the defects in the left knees were left empty. Half of these rabbits were randomly selected and given daily HBO treatment for 4 weeks. The HBO and HBO plus fibrin groups were comprised of these knees. The other half of the rabbits received no hyperbaric oxygenation, and the control and fibrin only groups were comprised of these knees. Four HBO-treated and 4 non-HBO-treated rabbits were sacrificed for histological study of cartilage repair at weeks 2, 4, 6, 8, and 12.

**Table 1:** Schedule of Histomorphological Analysis of Articular Repair After Surgery and Hyperbaric Oxygenation

Weeks after osteochondral surgery at harvesting of specimen	Rabbits (N = 40) Right knees - fibrin plugs; Left knees - no plugs	
	
	HBO Group, 20 rabbits	Non-HBO Group, 20 rabbits
	
	(Animals sacrificed in each group)	
2 weeks	4	4

4 weeks	4	4

6 weeks	4	4

8 weeks	4	4

12 weeks	4	4

Forty male New Zealand white rabbits with an age of 4 to 5 months old and weighing about 3 kg each were purchased from a licensed dealer. The animals were housed in our animal facility and were fed ad libitum. All animal procedures were performed according to the regulations of the authors' institute.

**Figure 1:**
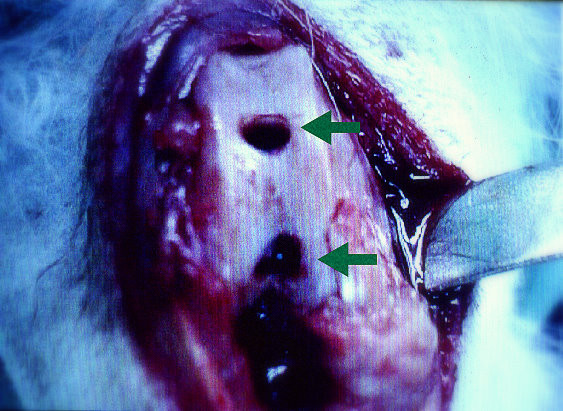
**Osteochondral defects were created over loaded (upper arrow) and unloaded (lower arrow) areas of the trochlea in the right femoral condyle**.

Each animal was anesthetized before surgery with an intramuscular injection of 10 mg/kg ketamine. Both knees were then arthrotomized using a medial parapatellar approach. Two full-thickness defects 3 mm in diameter and 3 mm in depth were drilled through the articular cartilage into bleeding subchondral bone in the trochlear groove of each femur. One hole was made in the center of trochlea that articulated with the patella, a weight-bearing surface; the other was made in the nonarticulated notch area, a non-weight-bearing surface (Figure 1). Both defects in each knee were blotted dry with a piece of gauze sponge to remove as much blood as possible before proceeding further.

**Figure 2:**
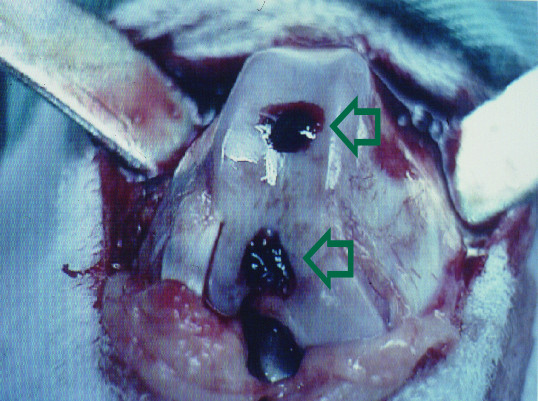
**The drilled holes were packed with exogenous fibrin clots (upper and lower hollow arrows)**.

The defects in the right knees were then packed with an exogenous fibrin clot that had been prepared from the animal intraoperatively. About 10 ml of whole blood was obtained from each animal and placed in a beaker until it clotted. These clots had a firm consistency, and could be easily handled. Fibrin clots were placed in the 2 right knee defects using fine-toothed forceps and packed with a blunt probe until the defects were filled flush to the surface of the adjacent cartilage (Figure 2). The patella was then reduced, and the joint capsule and skin were reapproximated with an interrupted suture of 3-0 nylon. A similar operation was used for the left knees, but the defects were left empty.

Postoperatively, the wounds were treated daily with neomycin ointment. In addition cefazolin 10 mg/kg was administered preoperatively, perioperatively, and daily for 48 hrs postoperatively. The limbs were not immobilized and the animals were allowed unrestricted movement in their cages immediately after recovery from anesthesia.

The day after surgery, half of the rabbits (n = 20) were randomly chosen to begin hyperbaric oxygen treatment, which was given 5 days a week for 4 weeks (20 treatments in total, except for 4 animals that were sacrificed for histological studies after 2 weeks and, hence, received only 10 treatments). These treatments were given in a hyperbaric animal research chamber (Model 2000, Mechidyne System Inc. Houston, TX, USA). In this compressed air chamber, 100% oxygen was delivered at 2.5 atmospheres absolute (ATA) for a duration of 120 min, using an intermittent schedule of 25 min of oxygen breathing and 5 min of air breathing.

The articular defects were evaluated at 2, 4, 6, 8, and 12 weeks after HBO therapy (Table 1). Four animals in each group (those treated and not treated with HBO) were euthanized with an overdose of phenobarbital for histological examination at each time point. After photographs were taken with a digital camera (RDC-2, Ricoh Co. Ltd., Tokyo, Japan), the distal portion of each femur was removed and fixed in 10% buffered formaldehyde. The specimen was then decalcified in 10% nitric acid. An osteochondral block including the 2 repaired defects was cut from the trochlea and embedded in paraffin. Five-μm-thick sections were cut in the sagittal plane, mounted on glass slides, and stained with either hematoxylin-eosin or Safranin O. Only the sections containing the repaired defects in the central trochlea (weight-bearing area) were then evaluated with a light microscope by using a histological grading scale (Table 2) [3]. At least 6 slices of each specimen (defect) were made and examined under different magnification powers of the microscope. To decrease the chance that the histological findings were merely incidental, we attempted to include as much of the normal area as possible beyond the repaired cartilage in each slide.

**Table 2:** Modified Histological Grading Scale for Defects in Articular Cartilage 3

Category	Score (points)
1. Filling of defect relative to surface of normal adjacent cartilage	
> 90%	0
75-90%	1
50-74%	2
25-29%	3
< 25%	4
2. Cellular morphology (percentage of chondrocytes)	
> 90%	0
75-90%	1
50-74%	2
25-49%	3
Mostly fibroblast-like cells	4
3. Surface architecture	
Normal	0
Slightly irregular	1
Fibrillation	2
Disrupted	3
4. Matrix staining with Safranin O	
Normal	0
Slightly reduced	1
Moderately reduced	2
Substantially reduced	3
None	4
5. Tidemark formation	
> 90%	0
75-90%	1
50-74%	2
25-49%	3
< 25%	4
6. Integration of repair tissue with adjacent articular cartilage	
Normal	0
Decreased cellularity	1
Small gap	2
Discontinuity	3
7. Percentage of new subchondral bone	
> 90%	0
75-90%	1
50-79%	2
25-49%	3
< 25%	4

Four groups of specimens were examined. Each group consisted of 20 knees. The left knees (with empty defects) of the non-HBO-treated animals were the control for the other 3 groups. The group with fibrin clots only was composed of the right knees of the non-HBO-treated animals. The left knees of HBO-treated animals were used to evaluate the effects of HBO alone on cartilage repair; the right knees of these animals served as the experimental group treated with both fibrin plus HBO.

All specimens were examined by the same 2 observers (LCP and ACC), both of whom were blinded to the treatments used. The grading scale used was a modification of the method of O'Driscoll [3,17,20,21] and was designed to evaluate subtle histological changes during repair, to reduce observer bias, and to allow quantitative comparisons between different experimental groups. Seven categories were used for histological assessment (Table 2). Categories 1 and 2 quantitatively and morphologically represented the degree of defect repair. Category 3 was designed to evaluate the surface architecture of the repair tissue. Category 4 addressed matrix production by staining for proteoglycan with Safranin O. Tidemark formation (category 5) and integration of repaired tissue with surrounding cartilage (category 6) were also evaluated. Category 7 addressed the repair of subchondral bone, with 100% replacement indicating complete regeneration of subchondral bone to the level of the original tidemark. All morphological changes and percentages were converted into histological scores [12]. The total score on this 7-category scale ranged from 0 (normal cartilage) to 26 points (no repair).

Mean scores for each time period were calculated from the average of the total scores of all 4 specimens in each group, and were expressed as mean ± standard deviation. The Kruskal-Wallis 1-way analysis of variance by ranks test was used to analyze differences between groups. When the Kruskal-Wallis test indicated a significant difference between groups, selective comparisons between the HBO+fibrin group and the other groups were performed using the Mann-Whitney rank sum test. A *P *value of ≤ 0.05 was considered to indicate statistical significance.

## Results

Gross examination of the surfaces of the defects showed that although the defects in the central, weight-bearing, area of the trochlea were completely covered by 2 weeks after surgery in all animals, it was not until 8 weeks after surgery that the defects in the peripheral, non-weight-bearing, area were completely covered in all animals.

**Figure 3:**
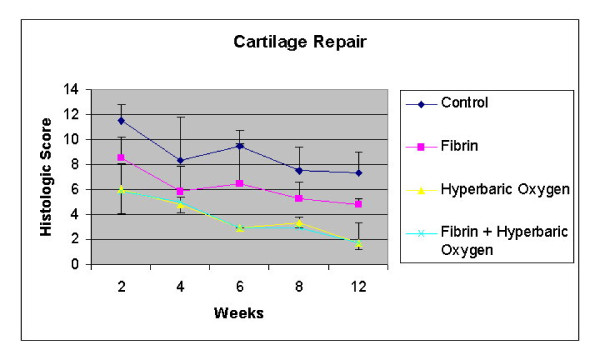
**Time course of repair of osteochondral defects in rabbit knees**. Complete healing is a histological score of "0". *Standard deviation is shown by the upper bars. ** Standard deviation is shown by the lower bars.

The data for each histological category at each time point for all 4 groups are shown in Table 3. Analysis of total histological score (our outcome measure for cartilage repair) with respect to time (Figure 3) showed that repair in the hyperbaric oxygen plus fibrin group was significantly faster and more complete than in the control and fibrin only groups; however, there were no such differences with the HBO only group. Also, except at 2 weeks after surgery, when the hyperbaric oxygen treatment had not yet been completed, the standard deviations for the HBO and HBO plus fibrin groups were noticeably smaller than those for the non-HBO-treated groups. In other words, recovery from osteochondral defects was slower and much more variable in the control and fibrin only groups than for those treated with hyperbaric oxygen. Our hypothesis was that healing would be better after treatment with HBO plus fibrin than with fibrin or HBO alone; therefore, our analysis only compared the HBO plus fibrin group with the other 3 groups. We did not analyze whether the HBO group (for which the histological data were almost identical to those of the HBO plus fibrin group) healed significantly faster than the control or fibrin groups.

**Table 3:** Histological Grading Scores

Time	Group (N = 4)*	Filling of Defect (Category 1)	Cellular Morphology (Category 2)	Surface Architecture (Category 3)	Matrix Staining (Category 4)	Tidemark (Category 5)	Integration (Category 6)	Subchondral Bone (Category 7)	Total Score	p Value^†^
2 weeks	C	1.5 ± 0.6	2.0 ± 0.0	1.8 ± 0.5	2.0 ± 0.0	2.3 ± 0.5	2.0 ± 0.0	2.0 ± 0.8	11.5 ± 1.3	0.021
	F	1.3 ± 0.5	1.5 ± 0.6	2.0 ± 0.0	1.0 ± 0.0	1.5 ± 0.6	1.3 ± 0.5	1.0 ± 0.0	8.5 ± 1.7	0.076
	H	0.8 ± 0.5	1.0 ± 0.8	1.3 ± 0.5	0.8 ± 0.5	1.3 ± 0.5	1.0 ± 0.0	1.5 ± 0.6	6.0 ± 2.1	0.655
	H+F	0.3 ± 0.5	1.0 ± 0.8	1.3 ± 0.5	1.0 ± 0.0	1.3 ± 0.5	1.0 ± 0.0	1.0 ± 0.8	5.8 ± 1.7	

4 weeks	C	0.5 ± 0.6	1.5 ± 1.3	1.5 ± 0.6	1.8 ± 0.5	1.5 ± 0.6	1.5 ± 0.6	1.8 ± 0.5	8.3 ± 3.5	p = 0.353^‡^
	F	0.5 ± 0.6	0.8 ± 1.0	1.0 ± 0.0	1.5 ± 0.6	1.0 ± 0.0	1.0 ± 0.0	0.5 ± 0.6	5.8 ± 2.1	
	H	0.0 ± 0.0	1.0 ± 0.0	0.8 ± 0.5	1.0 ± 0.0	0.8 ± 0.5	1.0 ± 0.0	1.3 ± 0.5	4.8 ± 0.6	
	H+F	0.0 ± 0.0	1.0 ± 0.8	1.0 ± 0.0	1.0 ± 0.0	1.0 ± 0.0	1.0 ± 0.0	0.0 ± 0.0	5.0 ± 0.8	

6 weeks	C	0.5 ± 0.6	1.8 ± 0.5	2.0 ± 0.0	1.5 ± 0.6	2.5 ± 0.6	1.3 ± 0.5	2.0 ± 0.0	9.5 ± 1.3	0.014
	F	0.5 ± 0.6	1.0 ± 0.8	1.3 ± 0.5	1.3 ± 0.5	1.3 ± 0.5	1.3 ± 0.5	1.5 ± 0.6	6.5 ± 3.1	0.014
	H	0.0 ± 0.0	0.0 ± 0.0	1.0 ± 0.0	0.0 ± 0.0	1.0 ± 0.0	1.0 ± 0.0	0.8 ± 0.5	3.0 ± 0.0	1.000
	H+F	0.0 ± 0.0	0.0 ± 0.0	1.0 ± 0.0	0.0 ± 0.0	1.0 ± 0.0	1.0 ± 0.0	0.5 ± 0.6	3.0 ± 0.0	

8 weeks	C	0.5 ± 0.6	1.8 ± 0.5	1.8 ± 0.5	1.5 ± 0.6	1.5 ± 0.6	1.0 ± 0.0	1.8 ± 0.5	7.5 ± 1.9	0.013
	F	0.3 ± 0.5	1.0 ± 0.0	1.0 ± 0.0	1.0 ± 0.0	1.3 ± 0.5	0.8 ± 0.5	1.3 ± 0.5	5.3 ± 1.3	0.013
	H	0.0 ± 0.0	0.3 ± 0.5	1.0 ± 0.0	0.0 ± 0.0	1.0 ± 0.0	1.0 ± 0.0	1.0 ± 0.0	3.3 ± 0.5	0.317
	H+F	0.0 ± 0.0	0.0 ± 0.0	0.8 ± 0.5	0.3 ± 0.5	1.0 ± 0.0	1.0 ± 0.0	1.0 ± 0.0	3.0 ± 0.0	

12 weeks	C	0.3 ± 0.5	1.3 ± 0.5	1.5 ± 0.6	1.3 ± 0.5	1.8 ± 0.5	1.3 ± 0.5	1.8 ± 0.5	7.3 ± 1.7	0.019
	F	0.3 ± 0.5	1.0 ± 0.0	1.0 ± 0.0	0.5 ± 0.6	1.0 ± 0.0	1.0 ± 0.0	1.3 ± 0.5	4.8 ± 0.5	0.017
	H	0.0 ± 0.0	0.3 ± 0.5	0.5 ± 0.6	0.3 ± 0.5	0.5 ± 0.6	0.3 ± 0.5	0.8 ± 0.5	1.8 ± 1.5	0.536
	H+F	0.0 ± 0.0	0.8 ± 1.0	0.5 ± 0.6	0.0 ± 0.0	0.3 ± 0.5	0.3 ± 0.5	0.5 ± 0.6	1.8 ± 0.6	

*Group: C = control; F = fibrin; H = hyperbaric oxygen (HBO); H+F = HBO+fibrin; ^†^A p value of 0.05 or less on the Mann-Whitney rank sum test was considered statistically significant for selective comparisons between the H+F group and other groups; ^‡^P value was 0.353 on the Kruskal-Wallis test, indicating that there was no significant difference between groups.

**Figures 4:**
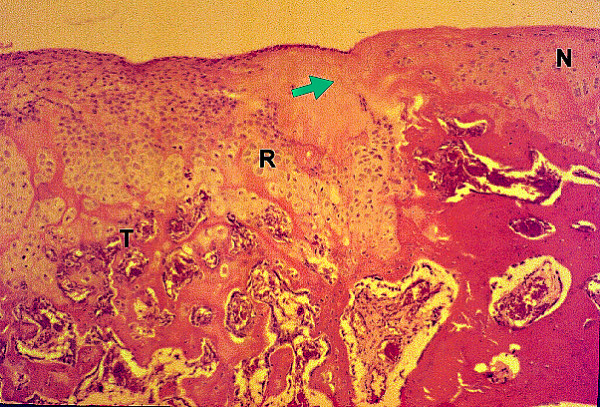
**Histology of osteochondral repair sites in the HBO plus fibrin group at 6 weeks**. Partail integration (arrow) of repair tissue (R) and adjacent normal cartilage (N) can be seen at 6 weeks. Tidemark (T) formation, subchondral bone formation, and repair of surface architecture are also noted. HE stain, original magnification × 25.

**Figures 5:**
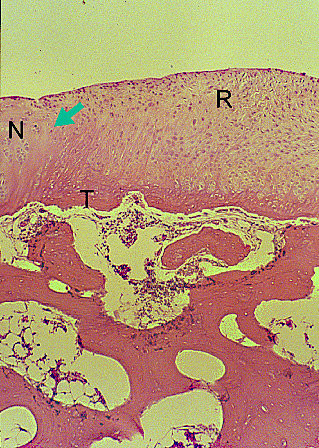
**Histology of osteochondral repair sites in the HBO plus fibrin group at 8 weeks**. Good integration (arrow) of repair tissue (R) and adjacent normal cartilage (N) can be seen at 8 weeks. Tidemark (T) formation, subchondral bone formation, and repair of surface architecture are almost complete. HE stain, original magnification × 25.

At 2 weeks, the HBO plus fibrin group exhibited significantly better filling (category 1) of defects (p = 0.021) than the control group, which had only sparse cellular infiltration in the repaired tissue at that time point. At 4 weeks, the HBO plus fibrin group showed complete filling (category 1) with good integration (category 6). At 6 weeks, significant differences in the histological grading in each category were noted between the HBO plus fibrin group (Figure 4) and the control (p = 0.014) and fibrin only (p = 0.014) groups. At 8 weeks, tidemark formation (category 5), subchondral bone formation (category 7), and repair of surface architecture (Figure 5) were almost complete in the HBO plus fibrin group, and significantly better scores were also noted in all grading categories for the HBO plus fibrin group, as compared with the control (p = 0.013) and fibrin only (p = 0.013) groups. Although cellularity at the repair/normal junction was decreased, there was good integration and continuity between the repaired tissue and normal cartilage.

**Figure 6:**
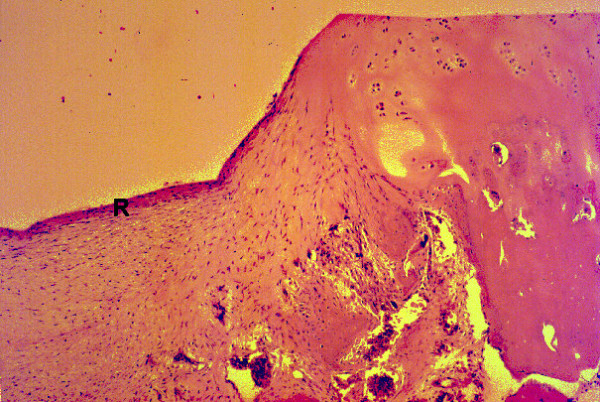
**HE staining of the control (non-HBO, non-fibrin) group show irregular fibrous repair (R)**. At 12 weeks, original magnification × 25.

**Figure 7:**
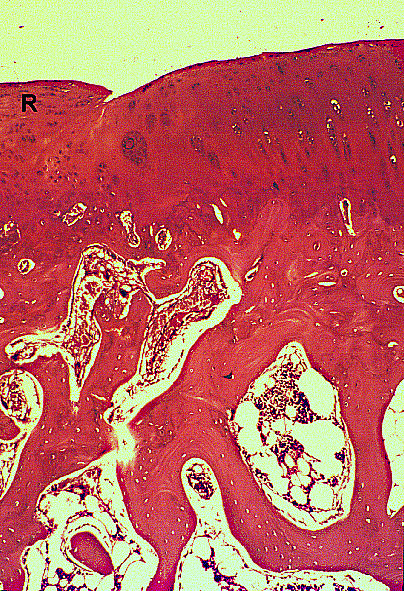
**Safranin O staining of the control (non-HBO, non-fibrin) group show irregular fibrous repair (R)**. At 12 weeks, original magnification × 25.

**Figure 8:**
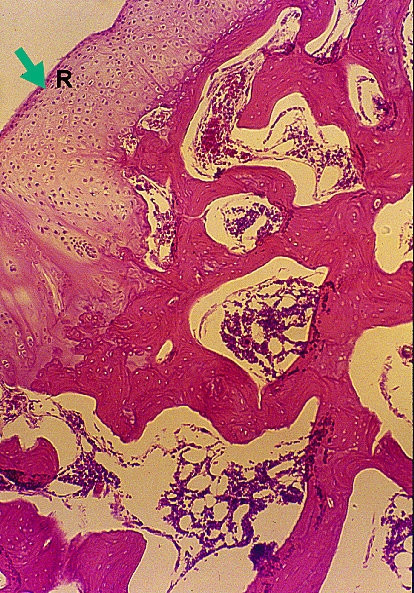
**HE staining of the experimental (HBO plus fibrin) group shows complete osteochondral repair (marked by an R, arrow)**. At 12 weeks, original magnification × 25.

**Figure 9:**
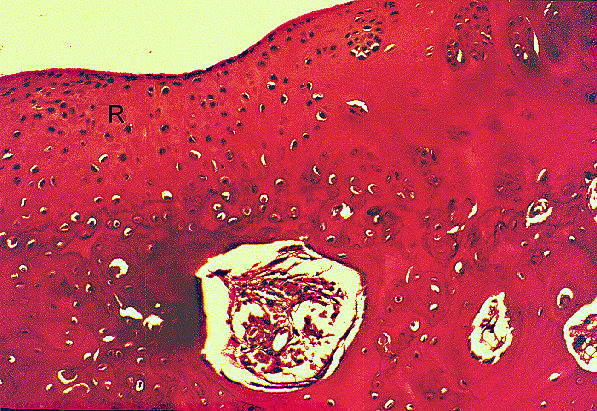
**Safranin O staining of the experimental (HBO plus fibrin) group shows homogeneous distribution of proteoglycan stain**. At 12 weeks, original magnification × 40.

At 12 weeks, the HBO plus fibrin group showed complete regeneration, proteoglycan staining that was similar to that of normal cartilage (category 4), and a homogeneous distribution of mature chondrocytes, while the control and fibrin groups showed only incomplete fibrous repair, and fibrillation and irregularity of the surface architecture (Figure 6, 7, 8 and 9). At this time point, the mean scores of the HBO plus fibrin group for each category were very close to 0 (normal cartilage) and, as at earlier time points, these scores significantly differed from those of the control (p = 0.019) and fibrin only (0.017) group.

There were no significant differences in histological grading scores between the 2 HBO groups (ie, the HBO only and HBO plus fibrin groups) at any time point.

## Discussion

Because fibrin and hyperbaric oxygenation have both been shown to improve wound healing, but by different mechanisms, we hypothesized that a combination of the 2 would have an additive effect on healing. However, this was not the case. HBO treatment was better than fibrin treatment, which in turn was better than no treatment. Nonetheless, adding fibrin clot treatment to hyperbaric oxygen treatment resulted in no significant additional benefit over hyperbaric oxygen alone.

In the current experiment, each treatment was used to treat defects in both the weight-bearing and non-weight-bearing areas of the cartilage surface. As was the case in previous research [12], the non-weight-bearing areas were slower to regenerate. In addition, our data confirm previous findings showing wide variation in the speed and extent of recovery in untreated cartilage defects [3]. This finding contrasted sharply with the uniformity seen in defects treated with hyperbaric oxygenation.

Although HBO clearly resulted in better repair than fibrin alone or no treatment, we did not test the repaired defects to establish whether the strength was normal. We also did not determine whether the collagen in the cartilage in the repair was type II or the inferior Type I variety. In addition, we did not conduct a longitudinal study to investigate whether the repair would deteriorate with time. Also, it is difficult to achieve integration of repair tissue with normal surrounding tissue. Although our subjective visual impression was that the repairs produced by HBO treatment were well integrated with adjacent cartilage, we have no quantitative data to support this.

Since vascular endothelial factors and neovascularization has been observed in the young growing cartilage [22], the improved repair process in the HBO groups might be due to the neovascularization triggered by HBO, which in turn facilitates osteochondral healing [13,18,19]. Hyperbaric oxygenation has long been known to cause angiogenesis and increase collagen synthesis. However, these mechanisms do not provide an explanation as to why adding fibrin, which acts by other mechanisms [17], fails to add to the effect of HBO.

Full-thickness cartilage repairs proceed in the following sequence: local bleeding and hematoma formation, migration of mesenchymal stem cells from the underlying bone, transformation of these cells into chondrocytes, proliferation of chondrocytes, synthesis of type I collagen, and filling of the defect with fibrocartilage rather than the physicochemically superior hyaline cartilage that is normally present [23]. This process is fuelled and directed by a variety of growth factors, some of which are known, others of which are not [11]. From a clinical perspective, similar processes may occur in acute injury, and in a chronic defect that has been trimmed and surgically refreshed in microfracture procedures [24]. However, cartilage formation may be disturbed in a late-treated articular defect because of altered joint homeostasis [25]. Whether HBO has similar positive effects on cartilage repair in older patients with longer existing cartilage defects is subject to future studies.

In addition to increasing neovascularization, hyperbaric oxygenation may directly affect the mixture of growth factors necessary for mesenchymal stem cell migration or the subsequent regenerative events. Unfortunately, the effects of HBO on tissue regeneration are difficult to determine because they do not all occur at the same oxygen concentrations. For example, in in vitro studies, glycosaminoglycan synthesis in bovine growth plate chondrocytes peaks at 21% O_2_, but proteoglycan aggregation is maximal at 3% O_2 _[26]; cell proliferation in rat calvarial bone cells is greatest at <9% O_2 _, but macromolecular synthesis peaks at >13% O_2 _[21]; chondrogenesis in periosteal organ culture is maximal at O_2 _concentrations of 12-15%, while inhibition of cartilage and type II synthesis occurs at very high (> 90%) and very low (< 5%) oxygen concentrations; and reactive oxygen species (which can be produced by hyperbaric oxygen) stimulate proteoglycan synthesis in chondrocytes at low concentrations and inhibit it at high concentrations [21]. We also do not know the optimal oxygen concentrations for regenerating cartilage under hyperbaric conditions. It has been estimated that HBO at 2 to 2.4 atmospheres will increase oxygen concentrations in bone 3-fold [13] and that 30-30 mm Hg O_2 _tension is needed for wound healing [13], but the actual concentrations of oxygen in regenerating cartilage are unknown. In vivo studies in chicks suggest that chondrocytes in endochondral growth cartilage are not hypoxic [27]; however, the current consensus is that cartilage and synovial fluid are hypoxic sites [1].

Fibrin clots in wound care in animal experimental models are believed to serve as a scaffold for repair of an osteochondral defect and to contain chemotactic and mitogenic factors that stimulate cellular elements crucial to tissue healing [17]. With the aid of a fibrin clot, experimental lesions healed more rapidly, and showed earlier subchondral bone formation, than did control lesions. However, in the presence of a fibrin scaffold alone, the entire cavity became populated with cells of metaplastic fibroblasts instead of mature chondrocytes, and the fibrocartilaginous repair tissue involved in defect filling (category 1) and surface architecture (category 3) ultimately resulted in similar scores for the control and fibrin-treated defects [13,17]. In normal cartilage, fibers have specific orientations, depending on their depth from the surface--those immediately beneath the surface are parallel to the surface, those at an intermediate depth are tangential to the surface, and those at the lowest depth, next to the bone, run perpendicular to the surface [20]. The presence of fibrin clots should lead to faster repair and a more normal surface architecture by providing an initial 3-dimensional matrix to which the regenerating chondrocytes fit into as they initiate collagen deposition. In the present study, however, the effect of fibrin on cartilage repair was modest and did not add to the effect of HBO.

HBO treatment in this study resulted in a clear improvement in cartilage repair and, unlike other treatments, is completely noninvasive. We do not know if the repaired cartilage is as strong as normal cartilage, or if it will deteriorate over time; nor do we know if HBO therapy will work with defects that do not extend to the bone (the majority of defects), where the mesenchymal stem cells are present. These partial-thickness defects do not regenerate. However, the results reported here do increase hope that a clinically noninvasive method to induce cartilage regeneration will be developed.

## Conclusions

In conclusion, our results show that hyperbaric oxygen treatment is clearly superior to no treatment in hastening cartilage repair and producing histologically superior cartilage. Packing the defects with fibrin clots was less effective than HBO, and produced no additional improvement when added to hyperbaric oxygenation.

## Competing interests

The authors declare that they have no competing interests.

## Authors' contributions

ACY conceived the idea of the study, performed part of the literature review, and contributed to the drafting of the manuscript. MSL performed part of the literature review and assisted in analyzing the data. SSL assisted in animal surgery and in manuscript drafting. LCP contributed to the interpretation of the light microscopic study. SWU contributed to manuscript editing. All authors have read and approved the final manuscript.
